# The Prognostic Impact of Tumor Border Configuration, Tumor Budding and Tumor Stroma Ratio in Colorectal Carcinoma

**DOI:** 10.5146/tjpath.2022.01579

**Published:** 2023-01-15

**Authors:** Lamia Sabry Aboelnasr, Hala Said El-Rebey, Asmaa Shams El Dein Mohamed, Asmaa Gaber Abdou

**Affiliations:** Department of Pathology, Faculty of Medicine, Menoufia University, Menofia, Egypt

**Keywords:** Colorectal cancer, Tumor budding, Tumor stroma ratio, Tumor border, Prognosis

## Abstract

***Objective:*** Tumor border configuration, tumor budding and tumor stroma ratio are reliable histopathological parameters that play a central role in the invasion-metastasis cascade. This study aimed to investigate the prognostic impact of these parameters and a new combined score in colorectal cancer.

***Material and Method:*** A cohort of 103 colorectal cancer surgical specimens was retrospectively evaluated for tumor border configuration, tumor budding and tumor stroma ratio using H&E sections. A combined risk score was then constructed to divide cases into low risk-tumors and high risk-tumors.

***Results:*** Infiltrating tumor border, high tumor budding, low tumor stroma ratio and high combined risk score were associated with positive lymph node involvement, presence of metastasis, high tumor grade, lymphovascular invasion, poor overall survival and short recurrence-free survival. Infiltrating tumor border, high tumor budding and high combined risk score were associated with advanced T stage. High tumor budding, and low tumor stroma ratio were associated with perineural invasion. Infiltrating tumor border was associated with increased tumor size and conventional adenocarcinoma, high tumor budding and low tumor stroma ratio. Low tumor stroma ratio was associated with high tumor budding. On multivariate survival analysis, tumor stroma ratio was found to be an independent predictor for overall survival and recurrence-free survival.

***Conclusion:*** Tumor border configuration, tumor budding, tumor stroma ratio and the newly constructed combined risk score are potential predictors of outcome in colorectal cancer patients, suggesting that their incorporation in the routine histopathological evaluation could be useful in determining the prognosis of colorectal cancer cases.

## INTRODUCTION

Colorectal cancer (CRC) is one of the most common cancers worldwide. According to GLOBOCAN 2020 data, CRC is the third most frequently diagnosed cancer in the world representing 10% of all cancer diagnoses (1). Surgical resection is the primary treatment modality for early stage CRC. The most effective postsurgical tool for assessing prognosis is the histopathologic analysis of the resected specimen including TNM-classification according to the American Joint Committee on Cancer (AJCC) (2). However, studies revealed that patients’ outcome may vary considerably even within the same tumor stage (3). Thus, recognition, standardization, and reporting of further histomorphological prognostic features are important clues for more accurate stratification and individualized therapeutic approaches.

Tumor border configuration (TBC) has been reported to have prognostic significance that is independent of stage (4). According to Jass, TBC is classified in a two-tier system as either infiltrating or pushing. Tumors with an infiltrating growth pattern often show signs of epithelial–mesenchymal transition (EMT), which can be identified histologically by the presence of “tumor buds” (5). Tumor budding (TB) can be defined as the presence of isolated single cells or small cell clusters of less than five cells at the invasive front of tumor (6). TB is another representative of EMT where the cells display migratory and invasive properties through losing intracellular and cell-matrix contacts mediated by E-cadherin (7). The recent dataset for histopathological reporting of CRC by the Royal College of Pathologists recommended TB reporting (8). Regarding its prognostic impact in CRC, some studies showed its poor prognostic role while others denied (9,10).

Importantly, stromal cells also actively participate in EMT process. They play a central role in cancer initiation and invasion-metastasis-cascade (11). Tumor stroma ratio (TSR) is an estimate of the proportion of epithelial and stromal cells. Studies have shown a strong association between high stromal content and poor prognosis in different cancer types (12).

TBC, TB and TSR are highly producible, reliable and convenient histopathological parameters. However, their universal acceptance as reportable factors has been held back due to lack of studies, variation in methods and controversial results. This study aimed to investigate the prognostic impact of TBC, TB, TSR and a new combined score in CRC.

## MATERIALS and METHODS

### Patients and Samples

This retrospective study included 103 primary CRC cases. Inclusion criteria were as follows: curative surgical colectomy with lymphadenectomy specimens that were diagnosed by histopahology as adenocarcinoma. Exclusion criteria were as follows: cases with incomplete clinicopathologic records, lost follow-up or cases that received neoadjuvant chemotherapy or radiotherapy. All cases that met these criteria, through the period between 2015 and 2019, were included. All formalin-fixed, paraffin embedded tissue blocks were retrieved from the archival material of the Pathology Department, Menoufia University.

This study was approved by the Ethics Committee of the Faculty of Medicine, Menoufia University, Egypt. Patient demographics, and data including tumor location and size were obtained from the original pathology reports.

### Histopathologic Evaluation

Four μm-thick sections were cut from all representative tissue blocks and all sections were stained by H&E. The mean number of evaluated tissue slides containing the tumoral areas was 4 (range 4-5) slides for each case and the selected slides contained at least 75% tumor tissue. All slides were re-evaluated independently by 2 experienced pathologists (L.S.A and A.S.E) for assessment of pathologic stage, histopathologic type, tumor grade, presence of lymphovascular invasion (LVI) and perineural invasion (PI) according to the 2019 WHO classification of tumours of the digestive system (2). Studied CRC cases included both conventional and mucinous adenocarcinoma cases. Regarding TBC, TB score and TSR, all slides were scanned to select the single most appropriate slide that highly met the recommended criteria for each parameter assessment as mentioned later.

### Tumor Border Configuration (TBC)

The H&E slide selected for TBC assessment was the one showing the part of tumor with greater depth of invasion (i.e. slides used routinely to assess T stage). According to Jass, an infiltrating TBC was defined as dissection of malignant growth in the form of irregular clusters or cords through the bowel wall with diminished desmoplastic stromal response (5). In contrast, margins were considered pushing when they were reasonably well circumscribed with a clear delineation of the tumor invasive front and absence of widely dissecting tumor glands in the muscularis propria or mesenteric adipose tissue (13).

### Tumor Budding (TB)

First, the H&E stained sections were examined with a ×10 objective lens, and the slide showing an area of the invasive margin with the highest density of tumor buds was subjectively selected (hot-spot sampling). Then, the number of tumor buds was counted in 10 HPFs, in a field that measured 0.785 mm2. In sections with less than 10 HPFs available, buds were counted in as many adjacent HPFs as possible, and the mean number of buds was calculated according to this number of examined fields. As recommended by the International Tumor Budding Consensus Conference (ITBCC), the TB score was reported by using a 3-tiered system (low, 0–4 tumor buds; intermediate, 5–9 tumor buds; high, 10 or more tumor buds) (6).

### Tumor Stroma Ratio (TSR)

The H&E slide representing the deepest invasive front was selected from each case. In case of more slides to be representative for the deepest invasive front, slides were scanned using the ×10 objective lens to select the area with the highest stromal percentage, which was considered decisive (14). Then, an area where both tumor and stromal tissue are present within the field was selected using a ×20 objective lens. Tumor cells had to be present at all borders of the selected field (north-east-south-west) as described by Huijbers et al. (15). TSR was estimated per microscopic field and scored into two groups as high TSR (low stroma as ≤ 50%) and low TSR (high stroma > 50%). Areas of necrosis, mucin, major vascular structures and muscle tissue were visually excluded from the scoring.

### Construction of A New Combined Risk Score (CRS)

Infiltrating TBC, TB score > 5 (median) and low TSR were categorized as risk items. Final categories were as follows: low risk-tumors with ≤ 1 risk item and high risk-tumors with > 1 risk items.

### Survival Data

Overall survival (OS) was calculated from the date of surgery to either the date of death or the last follow-up. Recurrence-free survival (RFS) was calculated from the date of surgery until the date of recurrence based on typical imaging appearance and evidenced by positive colonoscopic biopsy findings.

### Statistical Analysis

Statistical analysis was performed using SPSS software version 22.0 (IBM SPSS Inc. IL, USA). Analyses of associations between the assessed histomorphologic variables and other clinicopathological variables were carried out by using χ2-tests. The Kaplan–Meier method and log rank test were used for survival analysis. Cox regression analysis was used to perform multivariable analysis of TBC, TB and TSR. A p value less than 0.05 was considered significant. For inter-observer variability analysis, Kappa (K) values were generated, and agreement was reported as moderate, substantial, and almost perfect for Κ values of 0.41–0.60, 0.61–0.80, and 0.81–1, respectively (16).

## RESULTS

### Clinicopathologic Data of the Studied CRC (Table 1)

**Table 1:** Clinicopathological characteristics of studied colorectal cancer (CRC) cases, relationship with tumor border configuration (TBC) and tumor budding (TB) score.

		**TBC**				**TB score**				
	**CRC cases** **(n=103)** **n (%)**	**Pushing** **(n=34)** **n (%)**	**Infiltrating** **(n=69)** **n (%)**	**X2**	**p**	**Low** **(n=45)** **n (%)**	**Intermediate** **(n=35)** **n (%)**	**High** **(n=23)** **n (%)**	**X2**	**p**
**Age (year)**										
≤ 55 years	40 (38.8)	10 (25)	30 (75)	1.89	0.168	16 (40)	14 (35)	10 (25)	0.43	0.81
> 55 years	63 (61.2)	24 (38.1)	39 (61.9)			29 (46)	21 (33.3)	13 (20.7)		
**Gender**										
Male	37 (35.9)	11 (29.7)	26 (70.3)	0.281	0.596	17 (46)	12 (32.4)	8 (21.6)	0.121	0.94
Female	66 (64.1)	23 (34.8)	43 (65.2)			28 (42.4)	23 (34.8)	15 (22.7)		
Tumor location										
Proximal colon	38 (36.9)	10 (26.3)	28 (73.7)	4.57	0.102	15 (39.5)	11 (28.9)	12 (31.6)	4.11	0.39
Distal colon	39 (37.9)	11 (28.2)	28 (71.8)			16 (41)	16 (41)	7 (17.9)		
Rectal	26 (25.2)	13 (50)	13 (50)			14 (53.8)	8 (30.7)	4 (15.4)		
**Tumor size (cm)**										
≤ 5.98 (mean )	51 (49.5)	23 (45.1)	28 (54.9)	6.675*	0.009*	28 (54.9)	14 (27.5)	9 (17.6)	5.16	0.07
> 5.98 (mean )	52 (50.5)	11 (21.2)	41 (78.8)			17 (32.7)	21 (40.4)	14 (26.9)		
**T stage**										
Early (T1-T2)	25 (24.3)	16 (64)	9 (36)	14.33*	<0.001*	19 (76)	4 (16)	2 (8)	14.06*	<0.001*
Advanced (T3-T4)	78 (75.7)	18 (23.1)	60 (76.9)			26 (33.3)	31 (39.7)	21 (26.9)		
**N stage**										
Negative lymph node involvement	56 (54.4)	28 (50)	28 (50)	16.02*	<0.001*	29 (51.8)	21 (37.5)	6 (10.7)	9.70*	0.007*
Positive lymph node involvement	47 (45.6)	6 (12.8)	41 (87.2)			16 (34)	14 (29.8)	17 (36.2)		
**M stage**										
Mx	35 (34)	9 (25.7)	26 (74.3)	6.818*	0.033*	12 (34.3)	12 (34.3)	11 (31.4)	28.95*	<0.001*
M0	60 (58.2)	25 (41.6)	35 (58.3)			33 (55)	22 (36.7)	5 (8.3)		
M1	8 (7.8)	0 (0)	8 (100)			0 (0)	1 (12.5)	7 (87.5)		
**Histopathologic type**										
Conventional adenocarcinoma	89 (86.4)	24 (27)	65 (73)	10.81*	0.001*	39 (43.8)	31 (34.8)	19 (21.3)	0.42	0.8
Mucinous adenocarcinoma	14 (13.6)	10 (71.4)	4 (28.6)			6 (42.8)	4 (28.6)	4 (28.6)		
**Tumor grade**										
High	24 (23.3)	1 (4.2)	23 (95.8)	11.77*	<0.001*	5 (20.8)	7 (29.2)	12 (50)	14.68*	0.001*
Low	79 (76.7)	33 (41.8)	46 (58.2)			40 (50.6)	28 (35.4)	11 (13.9)		
**Lymphovascular invasion**										
Positive	31 (30.1)	5 (16.1)	26 (83.9)	5.71*	0.017*	10 (32.3)	8 (25.8)	13 (41.9)	9.83*	0.007*
Negative	72 (69.9)	29 (40.3)	43 (59.7)			35 (48.6)	27 (37.5)	10 (13.9)		
**Perineural invasion**										
Positive	20 (19.4)	3 (15)	17 (85)	3.64	0.056	5 (25)	6 (30)	9 (45)	7.812*	0.02*
Negative	83 (80.6)	31 (37.3)	52 (62.7)	1.89	0.168	40 (48.2)	29 (34.9)	14 (16.9)	0.43	0.81

The age of the studied cases ranged between 25 and 85 years with a median of 57 years and a mean± SD of 55± 13 years. The greatest dimension ranged between 2 and 21 cm with a mean± SD of 5.98± 3.36 cm and 6 cm as a median value.

### TBC, TB Score, TSR and CRS in Studied CRC Cases

**Figure 1:**
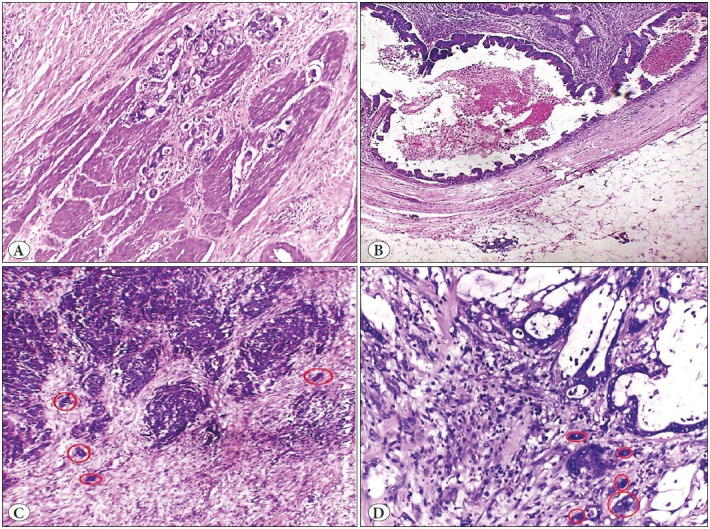
**A)** A case of colonic adenocarcinoma showing infiltrating tumor border configuration in the form of malignant growth dissecting muscularis propria (highlighted by a red circle) (H&E x100), **B)** A case of colonic adenocarcinoma with a pushing tumor border configuration (H&E x40), **C)** Foci of tumor budding at the invasive tumor margin (highlighted by red circles) (H&E x100), **D)** Foci of tumor budding at the invasive tumor margin (highlighted by red circles) (H&E x200).

**Figure 2:**
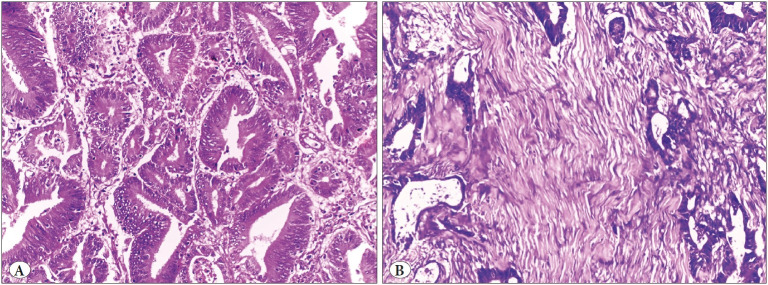
**A)** A case of colonic adenocarcinoma with malignant glands at 4 borders showing high tumor stroma ratio (TSR) (low stromal content < 50%) (H&E x200), **B)** Low TSR (high stromal content > 50%) (H&E x200).

Sixty-nine cases (67%) showed infiltrating TBC while 34 cases (33%) showed pushing TBC (Figure 1). Forty-five cases (43.7%) showed low TB score, 35 cases (34%) showed intermediate TB score and 23 cases (22.3%) showed high TB score (Figure 1). Sixty-seven cases (65%) showed high TSR, while 36 cases (35%) showed low TSR (Figure 2). Fifty-six cases (54.4%) had high CRS while 47 cases (45.6%) had low CRS.

### Inter-Observer Reproducibility of TBC, TB and TSR

Kappa value was 0.92, 0.83 and 0.72 for TBC, TB and TSR, respectively.

### Relationship Between Investigated Parameters (TBC, TB, TSR and CRS) and Other Studied Clinicopathological Parameters in CRC Cases

Infiltrating TBC was significantly associated with increased tumor size (p=0.009), advanced T stage (p<0.001), positive lymph node (LN) involvement (p<0.001), presence of metastasis (p=0.03), conventional adenocarcinoma (p= 0.001), high tumor grade (p<0.001) and LVI (p=0.017) (Table 1).

High TB score was significantly associated with advanced T stage (p<0.001), positive LN involvement (p=0.007), presence of metastasis (p<0.001), high tumor grade (p<0.001), LVI (p=0.007) and PI (p=0.02) (Table 1).

Low TSR was significantly associated with positive LN involvement (p=0.001), presence of metastasis (p<0.001), high tumor grade (p=0.006), LVI (p=0.005) and PI (p=0.009) (Table 2).

High CRS was significantly associated with advanced T stage, positive LN involvement, positive LVI (p<0.001 for each), presence of metastasis (p=0.026) and high tumor grade (p=0.005) (Table 2).

**Table 2:** Relationship of tumor stroma ratio (TSR) and combined risk score (CRS) with clinicopathological characteristics.

	**TSR**				**CRS**			
	**Low (n=36) n (%)**	**High (n=67)** **n (%)**	**X2**	**p**	**Low (n=47)** **n (%)**	**High (n=56)** **n (%)**	**X2**	**p**
**Age (year)**								
≤ 55 years	18 (45)	22 (55)	2.904	0.08	15 (37.5)	25 (62.5)	1.74	0.18
> 55 years	18 (28.6)	45 (71.4)			32 (50.8)	31 (49.2)		
**Gender**								
Male	15 (40.5)	22 (59.5)	0.79	0.37	17 (45.9)	20 (54.1)	0.002	0.96
Female	21 (31.8)	45 (68.2)			30 (45.5)	36 (54.5)		
**Tumor location**								
Proximal colon	11 (28.9)	27 (71.1)	3.49	0.17	20 (52.6)	18 (47.4)	1.35	0.507
Distal colon	18 (46.2)	21 (53.8)			17 (43.6)	22 (56.4)		
Rectal	7 (26.9)	19 (73.1)			10 (38.5)	16 (61.5)		
**Tumor size (cm)**								
≤ 5.98 (mean)	18 (35.3)	33 (64.7)	0.005	0.942	21 (41.2)	30 (58.9)	0.807	0.36
> 5.98 (mean)	18 (34.6)	34 (65.4)			26 (50)	26 (50)		
**T stage**								
Early (T1-T2)	6 (24)	19 (76)	1.74	0.187	20 (80)	5 (20)	15.7*	<0.001*
Advanced (T3-T4)	30 (38.5)	48 (61.5)			23 (29.5)	51 (70.5)		
**N stage**								
Negative lymph node involvement	12 (21.4)	44 (78.6)	9.87*	0.001*	35 (62.5)	21 (37.5)	14.07*	<0.001*
Positive lymph node involvement	24 (51.1)	23 (48.9)			12 (25.5)	35 (74.5)		
**M stage**								
Mx	18 (51.4)	17 (48.6)	15.72*	<0.001*	17 (48.6)	18 (51.4)	7.29*	0.026*
M0	12 (20)	48 (80)			30 (50)	30 (50)		
M1	6 (75)	2 (25)			0 (0)	8 (100)		
**Histopathologic type**								
Conventional adenocarcinoma	32 (36)	57 (64)	0.29	0.59	38 (42.7)	51 (57.3)	2.27	0.13
Mucinous adenocarcinoma	4 (28.6)	10 (71.4)			9 (64.3)	5 (35.7)		
**Tumor grade**								
High	14 (58.3)	10 (41.7)	7.52*	0.006*	5 (20.8)	19 (79.2)	7.75*	0.005*
Low	22 (27.8)	57 (72.2)			42 (53.2)	37 (46.8)		
**Lymphovascular invasion**								
Positive	17 (54.8)	14 (45.2)	7.715*	0.005*	3 (9.7)	28 (90.3)	23.1*	<0.001*
Negative	19 (26.4)	53 (73.6)			44 (61.1)	28 (38.9)		
Perineural invasion								
Positive	12 (60)	8 (40)	6.84*	0.009*	6 (30)	14 (70)	2.44	0.11
Negative	24 (28.9)	59 (71.1)			41 (49.4)	42 (50.6)		

### Relationship Between Investigated Parameters (TBC, TB, TSR)

**Figure 3:**
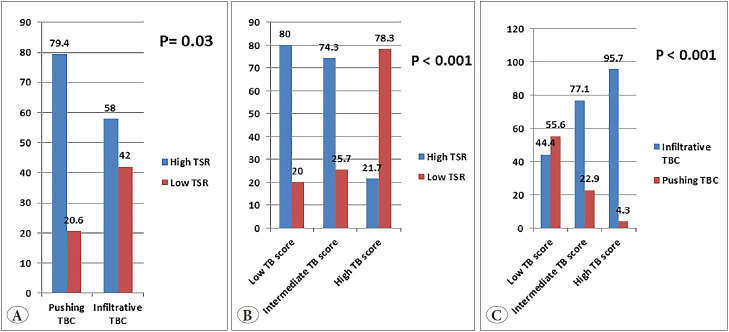
**A)** Infiltrating tumor border configuration (TBC) was significantly associated with low tumor stroma ratio (TSR) (p=0.03), **B)** Infiltrating TBC was significantly associated with high tumor budding (TB) score (p<0.001), **C)** High TB score was associated with low TSR (p<0.001).

Infiltrating TBC was significantly associated with low TSR (p= 0.03), and high TB score (p<0.001). Moreover, high TB score was associated with low TSR (p<0.001) (Figure 3).

### The Impact of Investigated Parameters (TBC, TB, TSR and CRS) on Survival

**Figure 4:**
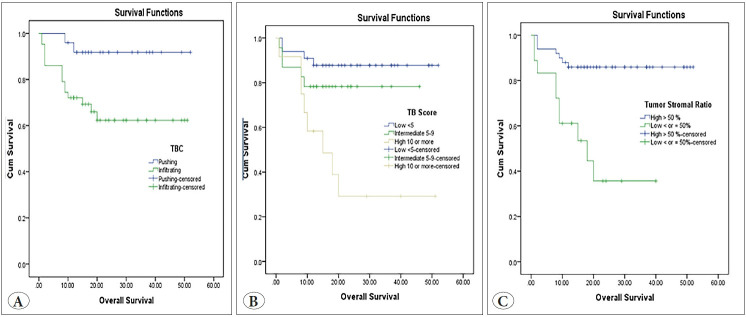
Kaplan-Meier survival curve demonstrating the impact of tumor border configuration (TBC) (p=0.015) **(A),** tumor budding (TB) score (p<0.001) **(B)** and tumor stroma ratio (TSR) (p<0.001) on overall survival **(C)**.

**Figure 5:**
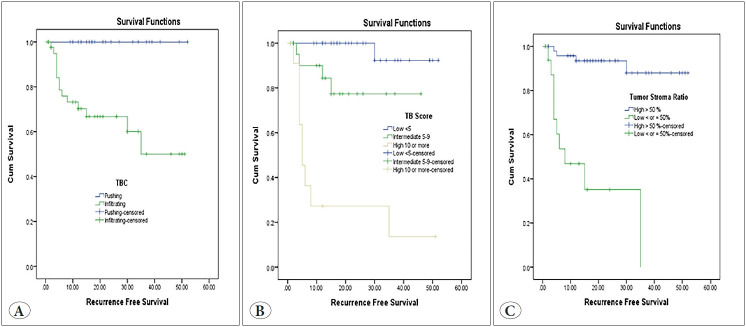
Kaplan-Meier survival curve demonstrating the impact of tumor border configuration (TBC) (p=0.001) **(A)**, tumor budding (TB) score (p<0.001) **(B)** and tumor stroma ratio (TSR) **(C)** on recurrence-free survival.

Univariate analysis of OS showed the bad prognostic impact of infiltrating TBC (p=0.015), high TB score (p> 0.001) and low TSR (p<0.001) (Figure 4). Infiltrating TBC (p=0.001), high TB score (p>0.001) and low TSR (p<0.001) were significantly associated with short RFS (Figure 5).

**Figure 6:**
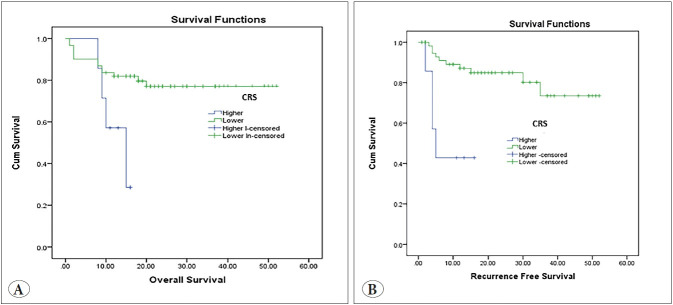
Kaplan-Meier survival curve demonstrating the impact of combined risk score (CRS) on overall survival (p<0.001) **(A)** and on recurrence-free survival (**B).**

High CRS was significantly associated with poor OS (p>0.001) and short RFS (p<0.001) (Figure 6).

On multivariate survival analysis, TSR was shown to be an independent predictor for OS and RFS (p=0.001) (p<0.001), respectively (Table 3).

**Table 3:** Multivariate COX regression analysis of overall survival and recurrence-free survival for the investigated parameters in studied CRC cases.

	**Overall survival**		**Recurrence-free survival**	
	**p**	**HR (95%CI)**	**p**	**HR (95%CI)**
Tumor Border Configuration (Infiltrating)	0.670	3.182 (0.236 – 18.902)	0.779	153.663 (0.695 – 435.624)
Tumor Budding Score (high)	0.204	9.215 (5.312 – 19.460)	0.192	5.021 (2.304 – 19.363)
Tumor Stroma Ratio (low)	0.001*	6.364 (1.410 – 21.720)	0.001*>	3.567 (1.452 – 8.817)

**HR:** Hazard ratio, **CI:** Confidence interval.

## DISCUSSION

The present study showed a poor prognostic impact of infiltrating TBC on OS and RFS, compared to pushing TBC. Infiltrating TBC had a significant association with adverse prognostic pathologic parameters such as large tumor size, advanced T stage, positive LN involvement, presence of metastasis, high tumor grade and LVI. These findings were in agreement with Morikawa et al. who observed that the infiltrating growth pattern was associated with worse prognosis among stage I-III CRC patients, independent of other clinical, pathologic, and molecular characteristics (17). Interestingly, the configuration of the invasive margin correlates with molecular alterations in CRC. Specifically, a well-demarcated, pushing tumor border is a feature frequently seen in MMR-deficient CRC-cases (18). In contrast, an infiltrating tumor border is significantly more frequent in tumors with activating *BRAF*-mutations (17). While MMR-deficient CRC generally has a favorable outcome, *BRAF* is an independent predictor of an aggressive clinical course (19). Data indicates that constitutive activation of *BRAF* may increase the migratory and invasive capacity of human colon cancer cells (20). This could contribute to the poor prognostic impact observed in CRC-cases with infiltrating TBC.

Moreover, the prognostic impact of TBC may refer to host-related factors that influence the appearance of the tumor border in CRC. Halvorsen and Seim described a marked absence of peritumoral inflammation in patients with an infiltrating TBC (21). In contrast, CRC-cases with a pushing border have a well-characterized association with dense peritumoral inflammatory infiltrate. Importantly, it is well-known that the density of peritumoral inflammatory response reflects the efficiency of anti-tumor host response, which may be a possible confounding factor of the good prognostic impact of pushing TBC (18,22).

Interestingly, unlike other studies that showed no correlation between TBC and histopathologic type of CRC, the present study showed a significant association between pushing TBC and mucinous colonic adenocarcinoma (MCA). This may be referred to the molecular profile stating that most of MCA occurs in patients with hereditary nonpolyposis CRC (HNPCC or Lynch syndrome) and thus represents high-level MSI (MSI-H) tumors which are known for their pushing margin configuration (23). Messerini et al. also reported a positive correlation between MSI-H MCA and expanding growth pattern (24). Reported prominent host immune response in MSI-H MCA may justify their decreased invasive potential represented in pushing TBC (25). In agreement with our observation, Hacking et al. reported that most of MCA cases had low TB score which was significantly associated with pushing TBC (26). Further research studies are recommended to investigate in depth the molecular characteristics of MCA in correlation to their histopathological features.

The present study demonstrated that high TB score was correlated with poor OS and short RFS. A high TB score showed a significant association with adverse prognostic pathologic parameters as advanced T stage, positive LN involvement, presence of metastasis, high tumor grade, LVI and PI. These findings were in agreement with Wyk et al. and Eriksen et al. who referred that to dedifferentiation of cells that tend to lose adhesion, dissociate and be more aggressive (9,10). There is a close relationship between TB and the process of EMT. In this transitional process, budding cells lose intracellular and cell-matrix contacts mediated by *E-cadherin*, migrate through the extracellular matrix, invade lymphovascular structures and form metastatic tumor colonies in lymph nodes and at distant sites (27,28).

The present study demonstrated the independent prognostic impact of TSR regarding both OS and RFS. This is in accordance with previous studies that reported the adverse prognostic impact of increased stromal component in both early disease and advanced colon cancer (9,14,29). Furthermore, low TSR was correlated with increased invasive and aggressive potential of CRC through its significant association with positive LN involvement, metastasis, high tumor grade, LVI and PI. Similar correlations were reported by Eriksen et al. and Zengin (9,30).

These findings may owe to the capability of stromal mesenchymal cells to orchestrate the invasion-metastasis-cascade (11). Several secreted molecular regulators of stromal cells have a pro-tumorigenic role. For example, upregulation of heat shock factor 1 (*HSF1*), Yes-associated protein 1 *(YAP1), Stromelysin 1* and stromal-derived exosomes have emerged as mediators of cancer progression through enhancing cancer cell motility, invasion, metabolic reprogramming and inducing cancer stem cell features (31).

As patients with stage II colon cancer have highly variable outcomes, TSR is a useful tool to select patients who are at risk of developing recurrence of disease or metastases. Huijbers et al. investigated the TSR next to the ASCO criteria; they found that the TSR improved the ASCO criteria and reclassified 14% of the patients as high‐risk. This suggests that adjuvant therapy might be considered in stage II patients with low TSR (15).

A significant association between TBC, TB and TSR was found with an infiltrating TBC related to increasing TB score and a higher fraction of stroma (low TSR). Eriksen et al. also observed a significant correlation between the mean number of buds and TSR with an increasing number of TB related to a lower TSR (9). Park et al. found an association between low TSR and the presence of an infiltrating invasive margin (32). Wang et al. reported that cases with high TB score had predominantly infiltrating TBC (33). This is in accordance with the consideration that the three parameters reflect the histopathologic translation of EMT where cancer cells assume a mesenchymal phenotype characterized by increased migratory capacity, invasiveness, increased resistance to apoptosis and increased production of extracellular matrix (ECM) components (28,34).

The integration of TBC, TB and TSR into an objective CRS model enhanced the prognostic impact of these parameters regarding OS and RFS. Furthermore, high CRS was significantly associated with advanced T stage, positive LN involvement, positive LVI, presence of metastasis and high tumor grade. These findings were in agreement with Dourado et al. who investigated a combined model of TB and TSR in oral squamous cell carcinoma (35). Interestingly, the CRS model has included both cancer cell features (TB, TBC) and stromal features (TSR). The present study is considered the first one to construct a combined model of TBC, TB and TSR and investigate its prognostic impact in CRC. Further studies are recommended to be conducted on larger cohorts for more validation.

Although assessment of TBC, TB and TSR was an easy method to apply, there were practical challenging difficulties. Peritumoral inflammatory response might be difficult to differentiate from TB, and may sometimes obscure the underlying budding. In such cases, immunohistochemistry staining for cytokeratin may help to highlight TB. Furthermore, in case of a stromal percentage at or around the cut-off point of 50%, consulting a second observer could be of help when in doubt. Overall, the inter-observer agreement was in a clinically useful and applicable range for the 3 parameters, ranging from substantial agreement in the setting of TSR to almost perfect in assessment of TBC and TB, in accordance with earlier studies using the same method (36,37). This high inter-observer agreement enhances the importance of adherence to a standardized scoring system and standardized protocol in the management of challenging settings during assessment (6,14).

In conclusion, TBC, TB score and TSR are highly reproducible, reliable and convenient parameters that could be easily assessed in H&E stained slides and included in routine histopathologic reports. The incorporation of these features into a CRS covering both epithelial and stromal features of tumor might be used to improve the stratification of CRC patients into low risk and high risk regarding their outcome.

## Conflict of Interest

All authors confirm that that there are no conflicts of interest.
